# Spatiotemporal Evolution of a Landslide: A Transition to Explosive Percolation

**DOI:** 10.3390/e22010067

**Published:** 2020-01-04

**Authors:** Kushwant Singh, Antoinette Tordesillas

**Affiliations:** School of Mathematics and Statistics, University of Melbourne, Parkville 3010, Australia

**Keywords:** explosive percolation, shear band, landslide, kinematics

## Abstract

Patterns in motion characterize failure precursors in granular materials. Currently, a broadly accepted method to forecast granular failure from data on motion is still lacking; yet such data are being generated by remote sensing and imaging technologies at unprecedented rates and unsurpassed resolution. Methods that deliver timely and accurate forecasts on failure from such data are urgently needed. Inspired by recent developments in percolation theory, we map motion data to time-evolving graphs and study their evolution through the lens of explosive percolation. We uncover a critical transition to explosive percolation at the time of imminent failure, with the emerging connected components providing an early prediction of the location of failure. We demonstrate these findings for two types of data: (a) individual grain motions in simulations of laboratory scale tests and (b) ground motions in a real landslide. Results unveil spatiotemporal dynamics that bridge bench-to-field signature precursors of granular failure, which could help in developing tools for early warning, forecasting, and mitigation of catastrophic events like landslides.

## 1. Introduction

Granular failure is not spontaneous. There exists a precursory failure regime in which patterns in motion emerge and evolve in space and time [1,2,3,4,5,6,7,8,9,10]. In natural field conditions, these patterns are further complicated by various multiphysics phenomena including seismic [11], rainfall [12], human activities [13,14], or a combination of these [6]. Quantitative knowledge of the spatiotemporal dynamics of these patterns is key to early detection of failure from the laboratory to the field. Unfortunately, despite the considerable efforts invested to the study of kinematics in granular systems, both of grain motions in the laboratory and ground motions in the field, a broadly accepted and useful bellwether for failure and its spatiotemporal statistical signature are still missing [3,5,7,15]. Filling this knowledge gap is crucial for developing effective diagnostic and forecasting tools for structural health monitoring (SHM) [4,8,10] and geohazard early warning systems (EWS) [12,13,16,17]. This research need has arguably become one of the most pressing issues in an era where remote sensing and imaging technologies are generating big data on motion and associated deformation continuously in real-time [10,18].

In this study, our goal is to develop a kinematic data-driven approach that can help fill this knowledge gap using the concept of critical transitions in percolation theory. Recent preliminary work [19] explored the process of percolation in time-evolving graphs embodying information on grain motions in a dense granular material during failure. That study uncovered evidence of explosive percolation in the state space of kinematics, but the study was essentially confined to the failure regime. By contrast, our interest lies in the pre-failure regime with a view towards applying percolation theory to find clues to the likely location and time of impending failure.

The concept of explosive percolation transition was first introduced a decade ago by Achlioptas, D’Souza, and Spencer (2009) [20]. In striking contrast to the continuous phase transition from classical percolation theory [21], the so-called “Achlioptas process” leads to an abrupt and discrete jump in the order parameter at the percolation transition—an “explosive” emergence of a system-spanning giant component. This dramatic change in percolation transition arises by introducing an element of competition in the Erdös-Rényi random graph construction process [21]. In the Achlioptas process, a pair of candidate edges, drawn at random, competes for selection. The winning edge is then chosen following a rule that systematically suppresses the growth of the largest cluster. In the short-term, the process promotes the growth of multiple coexisting connected clusters (or components) of comparable size. However, in the long-term the components become increasingly primed for a spontaneous merger that then precipitates an explosive, albeit delayed, critical transition to a system-spanning giant component. Although this new paradigm of percolation has attracted considerable attention in the literature, its applications to real-world systems are still being discovered [22].

Here, our interest lies not in the Achlioptas process per se, but in the outcome of this process from the perspective of a time evolving clustering point pattern in a given feature state space. In particular, recent studies have shown that collective motion develops in the precursory failure regime, where the granular body splits into subgroups, in each of which constituent grains move in near-rigid body motion [2,5,9]. When mapped to a graph where edges are assigned based on kinematic similarity, this collective motion leads to a modular structure comprising densely connected communities, in between which are bridge nodes of relatively sparse connectivity. These bridge nodes, which distinguish themselves by having high closeness centrality, can reliably predict the failure location in the early stages of the pre-failure regime. Encouraged by this, we herein adopt a connection rule that is also governed by kinematic similarity: nodes within a distance *r* of each other in kinematic state space are connected. Some investigations into explosive percolation have also used connection rules based on the similarity of relevant system features [23,24].

At each observed time state of the system, we characterize the evolving connectivity of the graph G(r,t) as *r* is increased incrementally. In this context, the sudden emergence of a system-spanning component arises when *r* reaches a critical value that quantifies the relative motion of the subregions of the system at the given time state. We summarize this with respect to the probability profiles p(r) versus *r*, where p(r) represents the probability that a given node belongs to the giant component: p(r) is close to zero (one) when G(r,t) is sparsely (densely) connected.

Our strategy rests on the existence of an intermediate or precursory failure regime when the system transitions from the stable to the failure regime, which can be characterized by a kinematic field manifesting the following spatiotemporal dynamics. (a) Intracluster similarity in motion increases as grains belonging to the same cluster begin to move in near rigid-body motion, at the same time as (b) intercluster similarity in motion decreases (i.e., intercluster separation in kinematic state space grows). We test this approach using data from simulations of two classic laboratory tests on granular failure, and a real data from ground-based radar monitoring of a landslide that develops in an operational open-pit mine. Our objective is twofold: (a) to determine whether the components in the resultant network can provide an early prediction of the failure location, and (b) to establish the extent to which the transition from continuous to explosive percolation with so-called powder keg properties in the sense of [25] can be used to identify a regime change point marking the time of imminent failure.

The remainder of this paper is organized as follows. We describe the data set in Section 2 and our method in Section 3. Key results are presented in Section 4 and then discussed in Section 5. Concluding remarks are given in Section 6. A glossary of key terms and symbols used in this paper is provided in the Appendix A.

## 2. Data

**Figure 1:**
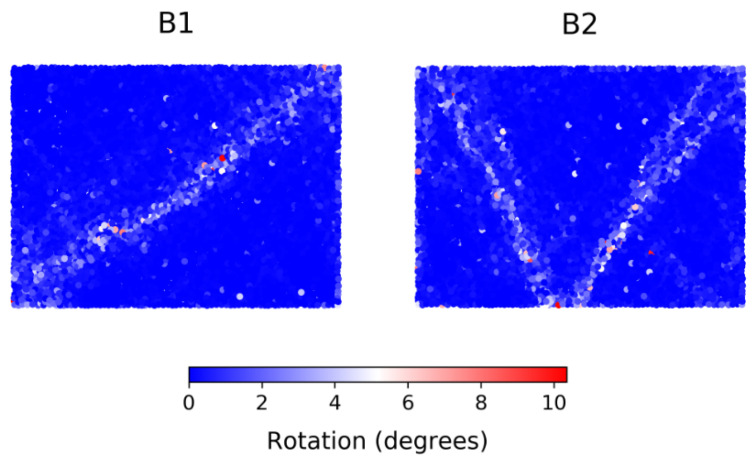
(Color online) Location of failure for samples B1 and B2, evident in the absolute accumulated rotations over the entire loading history.

We studied data labeled B1, B2, and Mine. These data sets are described in detail elsewhere: see the work in [26,27,28] for B1 and B2, and the work in [9] for Mine. B1 and B2 are from discrete element simulations of a well-studied assembly of initially homogeneous, densely-packed, polydisperse spherical grains, confined to planar motion. The samples, while identical initially, are submitted to two different biaxial loading conditions, resulting in two distinct failure evolution patterns. B1 (B2) is a displacement-driven compression in the vertical direction under constant confining pressure (constant volume). The failure of these initially homogeneous samples is realized through strain localization, with the ultimate persistent shear band pattern portraying: a single band extending diagonally from the bottom left corner to the top right corner of B1, and two shear bands in the shape of the letter V in B2 (Figure 1). Once fully formed, the shear band partitions the sample into near-rigid body (or underforming) components that can slide relative to each other along their shared boundary—the shear band itself. In the case of B2, the two shear bands interact and take turns in going from passive to active phase. During the passive phase, there is a stress build-up or “jamming” in the shear band; in the active phase, there is stress relief or “unjamming”, which is accompanied by the components on either side of the band undergoing relative sliding [28,29]. Shear band evolution in these samples has been studied using different techniques including those from: complex networks [27], spatial statistics [28], and dynamical systems [29]. In these past studies, particular attention was paid to quantifying energy dissipation and the correlated measure of nonaffine deformation, as well as identifying the mechanisms underlying dissipation including the birth-death evolution of structural load-bearing motifs (e.g., 3-cycles and subclasses of these, force chains, and vortices) [26,27,28,30].

**Figure 2:**
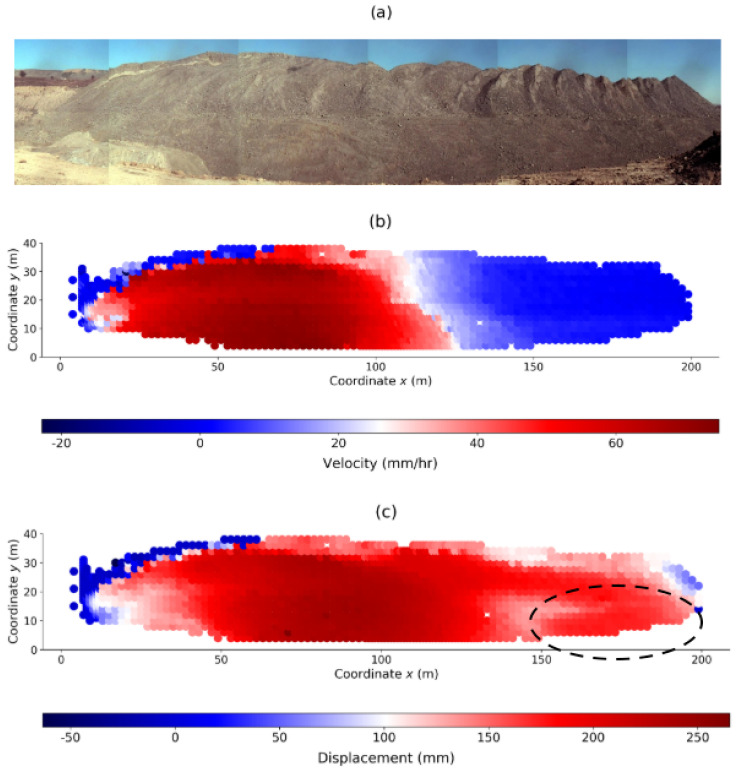
(Color online) (**a**) Monitored rock slope. (**b**) Velocity map at the time of peak velocity *t* = $t_{F}$ = 3568 = 13:10 15 June shows the location of failure (red zone). (**c**) Cumulative displacement map reveals significant movement in two locations at *t* = 3200 = 23:45 13 June: one to the west and another to the southeast corner (enclosed in dashed outline).

The Mine data is from monitoring of a rock slope in an operational open pit mine [9]. The mine operation, location, and year of the rockslide are confidential. The rock slope stretches to approximately 200 m in length and 40 m in height (Figure 2a). The slope stability radar technology that was deployed to scan the slope and continuously measure surface movement with sub-millimeter precision is described in [13,14]. The Mine data comprise surface displacement along a line-of-sight (LOS) from the stationary ground-based monitoring station to each observed location on the surface of the rock slope at every six minutes. The monitoring period is three weeks—from 10:07 31 May to 23:55 21 June—during which time series data were gathered from 1803 pixel locations across the entire slope at high spatial and temporal resolutions. On 15 June, a rockslide occurred on the western side of the slope with an arcuate back scar and a strike length of around 120 m (red zone in Figure 2b). A global average peak velocity of 0.56 mm/min (33.61 mm/h) was recorded at 13:10 15 June: we refer to this as the time of failure (or time of landslide) $t_{F}$. There is a “competing slide”, a second region of instability, to the southeast region (Figure 2c). This region intermittently developed large movements, but the instability was somehow arrested and movement slowed down the day before the collapse of the west wall. In this context, this region is sometimes referred to as a false alarm in the sense that it did not eventuate into a collapse [18]. The same phenomenon is observed in laboratory tests on real sand samples: competing localized failure patterns sporadically form (e.g., transient microbands and shear bands) just before failure but these later disappear to give way to the “winning” so-called persistent pattern of shear band(s) in the failure regime [1,2,9,28,31,32].

In all the systems above, instability patterns can be observed to develop in the precursory failure regime [9,26,27,28,31]. Past studies have demonstrated that such precursory patterns can be classified into one of two types: persistent versus transient [1,2,9,28,31,32]. The patterns in the former type persists in time with only minor changes to their spatial location and shape. By contrast, the transient patterns are sporadic and disappears just before failure, leaving behind the persistent patterns through to failure. In this study, we are interested in a regime change point $t^{*}$, which marks the time when failure becomes imminent. Our hypothesis is that two conditions must hold from $t^{*}$: (a) the shape and location of failure no longer change across time as the failure pattern becomes permanently incised in the system, and (b) and relative sliding or slip among the split components of the system initiates. In what follows, we develop a method that can quantify these conditions within the framework of explosive percolation theory.

## 3. Methodology

### 3.1. Algorithm Input and Network Construction

**Figure 3:**
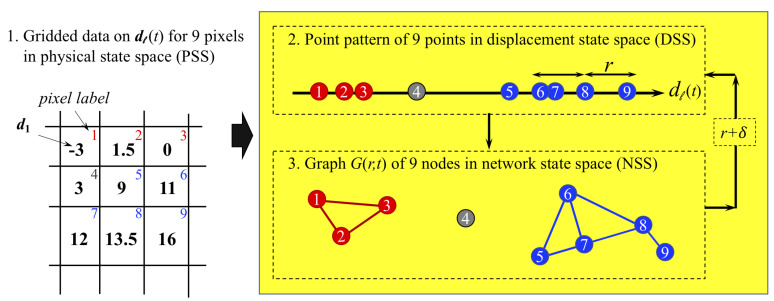
(Color online) Schematic of the method for constructing G(r,t) for N=9 pixels at a fixed time *t*. For each pixel *ℓ*, there exist a corresponding point whose coordinate in the displacement state space (DSS) is equal to the line-of-sight cumulative displacement $d_{\ell }\left(t\right)$, ℓ=1,2,…,9. Network G(r,t) is formed by connecting two nodes in NSS if their corresponding points in DSS are within a distance *r*. The evolving changes to the connectivity of G(r,t) is studied as *r* is increased.

The input to our analysis is a point pattern in an *n*-dimensional feature state space that evolves in time. The feature used here represents kinematics: displacements $u_{\ell }\left(t\right)$ of points ℓ=1,…,N at time states t=1,…T. At each time state, a corresponding graph G(r,t) is constructed in the network state space (NSS), where the nodes represent grains in B1 and B2 and pixels on the rock slope (Figure 3). An N×N matrix of weights is created, where the ij-th element describes the kinematic similarity between nodes *i* and *j*: $\|u_{i}-u_{j}\|$ for B1 and B2 where $u_{i}$ is a 2-dimensional displacement vector in the *x* and *y* directions; and $|u_{i}-u_{j}|$ for the Mine, where $u_{i}$ is a 1-dimensional displacement vector in the line-of-sight between the radar and a monitored pixel location on the slope surface. Two nodes, *i* and *j*, share a link if the ij-th element is less than *r*, where r>0 is a free parameter. We then study how G(r,t) changes as *r* increases from 0 by a constant and small increment δ. When *r* is small, only a few links exist and G(r,t) is a sparsely connected graph. As *r* increases, an increasing number of nodes are connected, so that eventually G(r,t) contains a system-spanning component. In previous studies of explosive transitions in real-world systems [23,24], the components in a network can be identified when links are added competitively. In this study we grow our network differently by using the parameter *r*. Consequently, our method identifies not just the components in a network but also quantifies their separation.

### 3.2. Algorithm Outputs

The outputs to our analysis at each t=1,2,…,T are (a) the order parameter p(r) evaluated over a range of increasing *r* values, (b) the components of the network $C_{k}$, and (c) a critical radius $r_{c,S}$. We will show that the components $C_{k}$ give an early prediction of the ultimate pattern and location of failure, whereas $r_{c,S}$ can be used to detect a regime change point $t^{*}$ that is indicative of imminent failure.

Let S(r) be the size of the largest connected component of G(r,t). The order parameter, p(r)=S(r)/N, characterizes the evolution of the network by the size of the largest component. If the growth of the largest component is suppressed, multiple coexisting components with sizes similar to that of the largest component will initially form in NSS [33]. Eventually, these components will coalesce explosively across multiple discontinuous jumps in p(r). Each discontinuous jump reflects the merger of a component to the largest cluster in the system, such that the number of such jumps is one less than the number of components in the system. Here, we are only interested in the major components of a network. Consequently, we limit our analysis to components with size at least αN, where α=0.1. We analyze the influence of the tuning parameter α on our results in Appendix B. The effect of a tuning parameter on the number of components that emerge in a network has also been analyzed in the context of a generalized Bohman–Frieze–Wormald (BFW) model [34] in [35].

If the network comprises multiple *M* components, denoted by $C_{j}$ for j=1,…,M, then $d(C_{j},C_{k})$ measures the kinematic separation of the two components by the minimum distance between two nodes in displacement state space (DSS): one in $C_{j}$, the other in $C_{k}$. The separation of a component, $d(C_{j})$, is then defined as the minimum distance of $C_{j}$ from another component in DSS:

(1) $$d\left(C_{j}\right)=\min_{k=1,\ldots ,M,\;\;j\neq k}d\left(C_{j},C_{k}\right).$$

**Figure 4:**
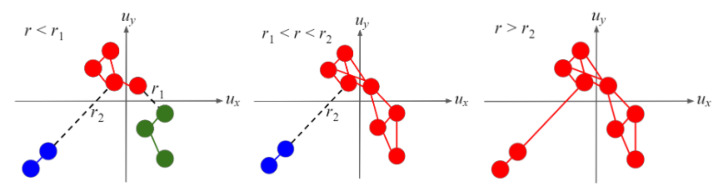
(Color online) Example of a clustering in DSS when M=3. Components merge at distinct *r* values. The blue component is an outlier and is far removed from the green and red components, therefore $C^{*}=C_{b}$ and $r_{c,S}=d\left(C^{*}\right)=r_{2}$.

The critical radius of separation is defined as the maximum separation of a component in DSS for time states, where there are at least two components: $r_{c,S}$ = $\max_{j=1,\ldots ,M}d\left(C_{j}\right)$ for M≥2. This maximally separated component, denoted by $C^{*}$, has the greatest kinematic separation of all other components in DSS: $d\left(C^{*}\right)=r_{c,S}$. $C^{*}$ is the last component that merges to form a system-spanning giant component and is reflected by the final discontinuous jump in p(r) that occurs at $r=r_{c,S}$. To illustrate, we depict in Figure 4 the case when M=3. The three components, $C_{b},C_{g},C_{r}$ (labeled according to their colors blue, green, and red, respectively), form and merge at distinct *r*. The separation values are $d\left(C_{g}\right)=d\left(C_{r}\right)=r_{1}$ and $d\left(C_{b}\right)=r_{2}$. As $r_{2}>r_{1}$, $r_{c,S}=r_{2}$ and $C^{*}=C_{b}$. If M=2 components emerge in NSS instead, then the separation of both components are equal: $d\left(C_{1}\right)=d\left(C_{2}\right)=r_{c,S}$ for components $C_{1}$ and $C_{2}$. In this case, an additional condition that is physically justified can be imposed to determine which of the two components should be denoted as $C^{*}$.

Components in DSS correspond to groups of grains (pixels) in physical state space (PSS). The collection of pairs of grains (pixels) from distinct groups in PSS that are physically in contact with each other forms a shared boundary. This boundary is a set of continuous lines or arcs if the components in DSS partition the system into distinct clusters in PSS. Therefore, the relative motion of the two groups of grains (pixels) on opposite sides of a shared boundary is quantified by $d(C_{j},C_{k})$, where $C_{j}$ and $C_{k}$ are their respective components in DSS. Naturally, $C^{*}$ induces a boundary in PSS where the relative motion of the two groups of grains (pixels) on opposite sides is maximal. In the next section, we show that the regime change point to imminent failure, $t^{*}$, is governed by two conditions: (a) the spatiotemporal persistence of $C^{*}$, and (b) a sharp increase in the motion of $C^{*}$ relative to the rest of the system. Physically, the former can be interpreted as the condition when the pattern of failure becomes incised in the system, whereas the latter condition is the onset of slip or sliding of $C^{*}$ relative to the rest of the system.

## 4. Results

### 4.1. Prediction of Location of Failure for B1 and B2

**Figure 5:**
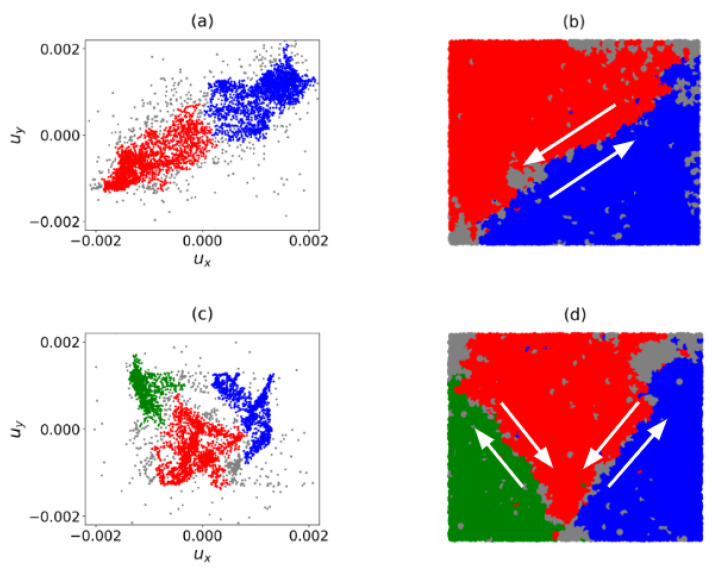
(Color online) Two components in B1 at pre-failure (t=65) (**a**) in DSS and (**b**) in PSS. Three components in B2 at pre-failure (t=70) (**c**) in DSS and (**d**) in PSS. Arrows depict relative motion along shared boundary.

We observe a precursory failure pattern in DSS in the precursory failure regime, namely, two components emerge in B1 and three in B2 (Figure 5a,c). When mapped back to PSS, these components partition the B1 and B2 samples into two and three subregions, respectively (Figure 5b,d). For both B1 and B2, the components are comparable in size, similar to the powder keg mechanism described in Friedman and Landsberg [25]. Comparing with Figure 1, it is evident that the shared boundaries of the components in the nascent stages of the precursory failure regime provide a reliable predictor of the ultimate location and pattern of failure.

In relation to previous studies, such precursory failure patterns can be classified into two types: transient versus persistent. Transient patterns form intermittently and then disappears at the onset of failure: for example, microbands [32]. By contrast, those in the latter persists in time with only minor changes to its spatial location and shape [1,2,3,5]. Of these, the persistent patterns provide a reliable early indicator of the location of the so-called ”persistent shear band” that governs the failure regime [1,2,3,5]. Note that the patterns in Figure 5 are only shown for a single time state. To establish the spatiotemporal persistence of these, we apply two measures: (a) the accumulation over a period of time states of the boundary location induced by $C^{*}$, characterized by the maximum relative motion of grains on opposite sides of this boundary, and (b) the Jaccard similarity index defined as follows,

(2) $$J_{t}=\frac{\left|A\cap B\right|}{\left|A\cup B\right|}$$

where *A* and *B* represent the set of nodes of $C^{*}$ in G(r,t) at two consecutive time states t−1 and *t*, respectively [36]. As $J_{t}$ is measured relative to the location of the maximally separated component $C^{*}$, a value of $J_{t}=1$ means $C^{*}$, at two consecutive time states, is exactly co-located in PSS. Note that $J_{t}$ depends on the existence of an $r_{c,S}$ value, as $d\left(C^{*}\right)=r_{c,S}$. Therefore, $J_{t}$ is only defined for time states where M≥2 components emerge in the sample.

**Figure 6:**
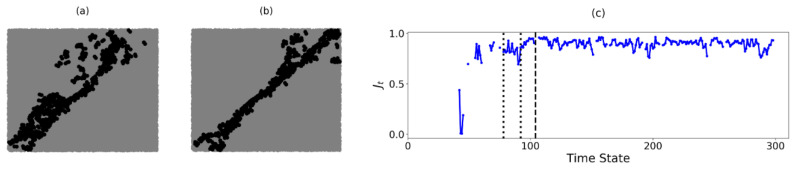
(Color online) Accumulation of the predictions of the shear band location in PSS (in black) for B1 from (**a**) t=78 to t=91 and (**b**) from t=92 to t=104. (**c**) Evolution of the similarity of $C^{*}$ in B1 with time. The dotted and dashed vertical lines represent the time states t=78, t=92 and $t_{F}=104$, respectively.

In B1, the two components, $C_{b}$ and $C_{r}$ (the blue and red components in Figure 5a respectively), are separated from each other by $r_{c,S}$: $d\left(C_{r}\right)=d\left(C_{b}\right)=r_{c,S}$. Both components share a boundary along the forward diagonal in the sample. In the B1-B2 samples, our key focus lies in identifying a persistent shear band pattern in the pre-failure regime from the boundary induced by $C^{*}$ in PSS. As a result, $C^{*}$ can be chosen to be either $C_{b}$ or $C_{r}$ as they lead to the same shear band prediction in B1. In Figure 6a,b below, the boundary of $C^{*}$ in B1 is accumulated across two periods of time states: (a) t=78 to 91 and (b) t=92 to 104, determined from the $J_{t}$ values in B1. Figure 6c shows that $J_{t}$ increases close to 1 at approximately t=78 and persists up to failure, with a small dip to $J_{t}\approx 0.7$ at t=91. This slight decrease in $J_{t}$ can be observed in the difference in the accumulated shared boundary in Figure 6b relative to Figure 6a: there is a marginal clockwise rotation of the accumulated shear band location about the bottom left corner of the sample. From t=92 to t=104, the predictions become concentrated along the forward diagonal in B1, indicating that the shear band becomes incised in the sample during this time period.

**Figure 7:**
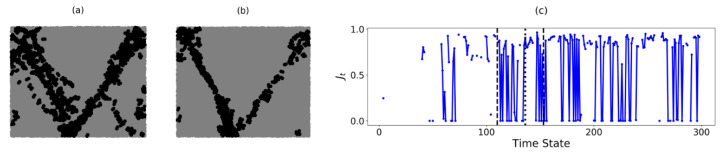
(Color online) Accumulation of the predictions of the shear band location in PSS (in black) for B2 from (**a**) t=110 to t=135 and (**b**) from t=136 to t=153. Note the higher density of black dots on the left (backward-inclined) band in (**b**). (**c**) Evolution of similarity of the boundary of $C^{*}$ in B2 with time. The dotted and dashed vertical lines represent the time states t=110, t=136 and $t_{F}=153$, respectively.

In B2, $C^{*}$ is a unique component at time states where three components emerge. Similar to B1, the boundary of $C^{*}$ in B2 is accumulated over two periods of time states: (a) t=110 to t=135 and (b) t=136 to t=153 (Figure 7a,b). In Figure 7c, the $J_{t}$ values appear infrequently prior to t=110. This is succeeded by rapid fluctuations in the $J_{t}$ values. Of the three components present: $C_{b}$, $C_{g}$ and $C_{r}$ (the blue, green and red components in Figure 5c respectively), $C^{*}$ alternates between $C_{b}$ and $C_{g}$ at different time states of the pre-failure regime. This results in the formation of two distinct shear bands, with the left (right) shear band corresponding to the boundary of $C^{*}$ when $C^{*}=C_{g}\left(C_{b}\right)$. Note that both boundaries are not always present in B2. As discussed in previous studies, not all shear bands are active at the same time: they take turns going from an active state (when the shear band unjams) to a passive state (when the shear band jams) and vice versa [28]. When $J_{t}=0$, the component $C^{*}$ switches from one side of the B2 sample to the opposite side (i.e., from $C_{g}$ to $C_{b}$ or vice versa). The fluctuations in $J_{t}$ thus indicate that the order in which the three components merge frequently changes, even though the failure pattern in B2 remains in the same spatial location. Between t=110 and t=135, the similarity index fluctuates between 0 and a maximum value of only $J_{t}\approx 0.8$. As such, the location of each shear band shifts over time, resulting in highly varied predictions seen in Figure 7a. Additionally, microbands [32] can be observed in the bottom right corner of the sample. From t=136 onward, the microbands disappear leaving only the two shear bands: $J_{t}$ is either 0 or much closer to 1. This implies that the two boundaries of $C^{*}$ in Figure 7b now persist in the same location (with the left boundary of $C^{*}$ or left shear band unjamming more often). Equivalently, the failure pattern of two bands in the shape of the letter V has become incised in B2.

### 4.2. Identification of Regime Change Point for B1 and B2

**Figure 8:**
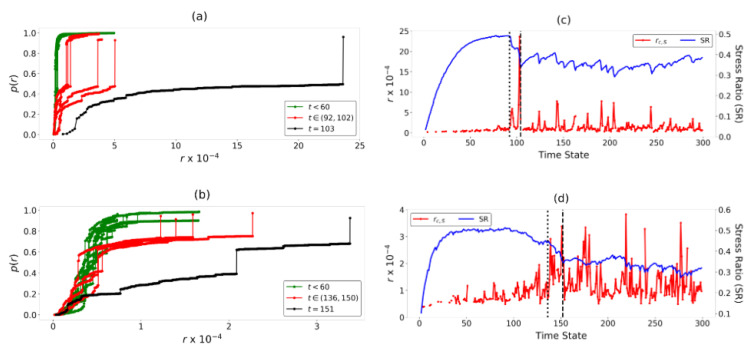
(Color online) Transition from continuous to explosive percolation at failure. Evolution with *r* of p(r) for samples (**a**) B1 and (**b**) B2 at various time states. Evolution with time state of stress ratio and $r_{c,S}$ for samples (**c**) B1 and (**d**) B2. Dotted and dashed vertical lines mark the regime change point ($t^{*}=92$ for B1 and $t^{*}=136$ for B2) and the start of the failure regime where the stress ratio fluctuates about a near constant value ($t_{F}=104$ for B1 and $t_{F}=153$ for B2), respectively.

We quantify the kinematic separation of the components in B1-B2 using the order parameter p(r) and critical radius $r_{c,S}$. The evolution with time of the p(r) vs. *r* profiles is shown in Figure 8a,b. The number of components that emerges at time *t* is evident in the number of discontinuous jumps in p(r). At the early time states, when the impending failure location is first predicted, we observe that the transition to a system-spanning component occurs at a small value of *r*. Consequently, the critical radius $r_{c,S}$ (Figure 8c,d) is also small at these time states. Closer to failure, a long plateau in p(r) develops during which increases in *r* merely lead to the addition of no links or intra-component links in G(r,t). An inter-component link finally forms at a large value of $r=r_{c,S}$, where $C^{*}$ merges to form a system-spanning component. This increasingly delayed transition is similar to what is observed in explosive transitions [33]. The long plateau in p(r) manifests as a sharp burst to a peak in $r_{c,S}$ at $t_{F}$. In B1, $r_{c,S}$ increases significantly at t=92 and remains relatively large for majority of the time states prior to the onset of failure at $t_{F}=104$. Similarly, in B2, an increase in $r_{c,S}$ values is observed at t=136 up to the onset of failure at $t_{F}=153$. As such, the time states $t^{*}=92$ and $t^{*}=136$ are the respective regime change points in B1 and B2. From $t^{*}$, the failure patterns become etched in the samples enabling relative sliding of $C^{*}$ with respect to its adjacent component.

As $r_{c,S}$ corresponds to the maximum kinematic separation of components in DSS, it quantifies the extent to which the clusters of grains in PSS move in opposing directions as near-rigid-bodies along the predicted shear band location. Therefore, it is not surprising that this measure of relative sliding between components correlate with energy dissipation in B1 and B2, as quantified in [26,28]. In B1, the two sharp bursts in $r_{c,S}$ and t=94 or t=103 represent two major slips between the two components along the predicted shear band location in B1. In B2, multiple peaks in $r_{c,S}$ between $t^{*}$ and $t_{F}$ suggest regular slips between the components: these occur along each of the two shear bands at different time states. We also note that previous studies of B1 and B2 [28] have shown that the shear band in B1 is less dense with lower average grain coordination number at failure than those in B2. This results in a higher degree of relative rigid-body-like motion between the two parts of the sample which explains the higher peak $r_{c,S}$ values in B1 compared to B2.

### 4.3. Prediction of Location of Failure for the Mine

Unlike the controlled and idealized loading conditions of the B1–B2 samples, the operational open pit mine was subjected to various perturbations such as mine blasting and weather changes. However, details of these external stimuli were not available. In this context, this study explores the extent to which meaningful information on impending failure can be uncovered from monitoring displacement data alone using the concept of explosive percolation. At this juncture, it is instructive to consider an additional kinematic feature state space, a 1-dimensional velocity state space (VSS), that can be easily derived from the given displacement data. A motivation for this is that velocity is considered widely in forecasting the time of failure in landslide monitoring [18]. Note that in the B1–B2 samples, the constant time intervals means that the velocities and displacements of the grains are proportional. Therefore, DSS is equivalent to VSS. This is not the case for the Mine since measurements at distinct pixel locations on the rock slope are taken along a line-of-sight: displacements are calculated with respect to a reference position while velocities are calculated with respect to the previous time state.

**Figure 9:**
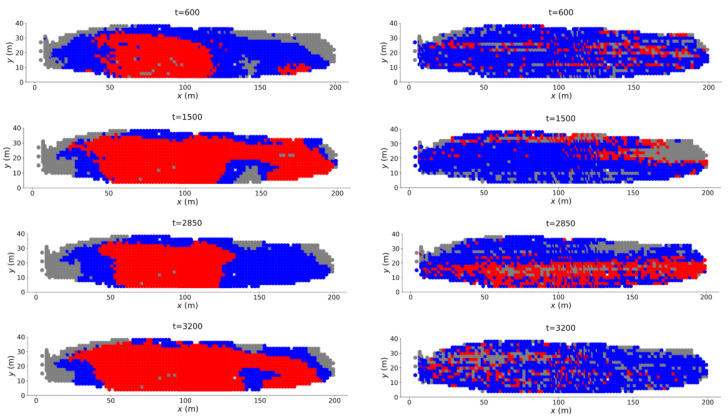
(Color online) Location of $C^{*}$ (red zone) in the Mine at multiple time states from t=600 to t=3200 obtained from the feature state space DSS (**left column**) and VSS (**right column**) in the early stages of the precursory failure regime.

As our aim is to predict the location of the rockslide (Figure 2), we are interested in M=2 components with the expectation that the failure region is that which exhibits anomalously high cumulative motion and rate of motion close to the time of failure. Thus, we assign $C^{*}$ to be that component with the higher mean cumulative displacement (velocity) in DSS (VSS). The predicted failure region from $C^{*}$ in DSS is shown in red at representative time states in Figure 9 (left column). In the early stages of the precursory regime, the prediction of the failure zone from DSS captures much of the west wall that ultimately collapses, along with the false alarm region to the southeast corner. As time advances, the red zone along the west wall persists, whereas that to the southeast corner appears intermittently. These two unstable zones interact which complicates the dynamics of the precursory failure regime [9,31]. As noted in [9], instabilities from the west shift to the middle, closer to the southeastern region when this is active. In fact, for most of these time states, these unstable zones combine to form one of the emerging components (e.g., at t=1500 and t=3200 in Figure 9 (left column)), consistent with Figure 2c. On the other hand, the location of $C^{*}$ in VSS is uninformative and corresponds to a group of pixels that is scattered in PSS (red zone in Figure 9 (right column)) at all time states in the early pre-failure regime. The group of pixels do not form a continuous separating boundary that is indicative of impending failure. The feature state space VSS can give misleading results in the early stages of the precursory regime, as transient false alarm regions can distort rates of motion by increasing at intermittent time periods only to slow down and stabilize later close to failure. This can be further compounded by using the spatially averaged velocity of pixels to identify $C^{*}$ in the early stages of the pre-failure regime.

To quantify the persistence of $C^{*}$ and distinguish between the persistent and transient regions, we introduce two similarity measures in our analysis: $J_{t}^{\left(1\right)}$ measures the similarity of the component $C^{*}$ in DSS across consecutive time states, whereas $J_{t}^{\left(2\right)}$ measures the geographical similarity of $C^{*}$ obtained separately from each kinematic feature state space, DSS and VSS, at the same time state. Note that in the terminal phase of the precursory regime, we can expect the failure location to portray not only higher cumulative displacements across time, but also significantly higher velocities compared to the stable zone. Moreover, as discussed earlier in Section 2, previous studies have shown that the transient patterns fade away close to failure leaving behind the persistent pattern that prevails through to failure [9,26,27,28,31]. Thus, altogether, these conditions imply that $C^{*}$ from DSS should become invariant in time while simultaneously converging to $C^{*}$ from VSS—resulting in a rise in both $J_{t}^{\left(1\right)}$ and $J_{t}^{\left(2\right)}$ to their maximum of 1.

**Figure 10:**
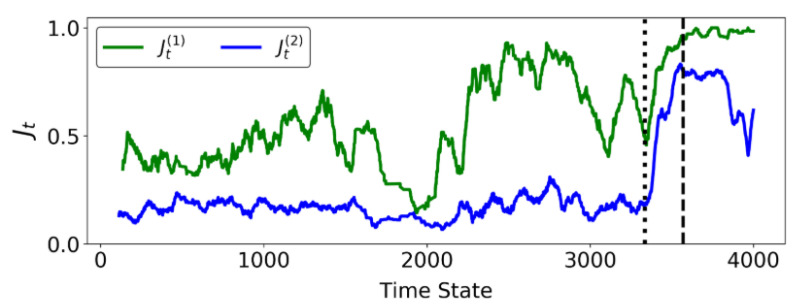
(Color online) The evolution with time of similarity ratios. Dotted and dashed vertical lines refer to time states t=3333 and $t_{F}=3568$ respectively.

**Figure 11:**
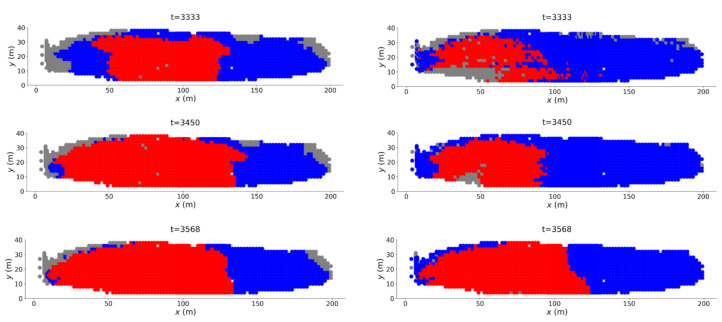
(Color online) Location of $C^{*}$ (red zone) in the Mine at multiple time states from t=3333 to t=3568 obtained from the feature state space DSS (**left column**) and VSS (**right column**).

In Figure 10, $J_{t}^{\left(1\right)}$ initially fluctuates between 0 and 1 reflecting the intermittent formation of the instability to the southeast: recall $C^{*}$ in Figure 9 (left column) most notably from t=2800 to 3000. Eventually, at t=3333, we observe a final increase in $J_{t}^{\left(1\right)}$ with only the pattern along the western wall remaining in the prediction. This transition at t=3333 is unambiguously confirmed in the sharp increase of $J_{t}^{\left(2\right)}$ at this time. The regime change point at t=3333 can be visualised in Figure 11. From t=3333, the transient pattern to the southeast disappears from $C^{*}$ in DSS (just as microbands to the bottom right corner in B2 have faded away (Figure 7a,b). The region along the west wall consolidates and slowly moves leftward before converging to the actual failure location at $t_{F}=3568$. The failure location is also present in $C^{*}$ from VSS, and coincides with that from DSS. In summary, our results here suggest that although we can predict the failure location early in the pre-failure regime using DSS, the more reliable indicator of imminent failure is the colocation in PSS of the critical components $C^{*}$ from DSS and VSS.

### 4.4. Identification of Regime Change Point for the Mine

**Figure 12:**
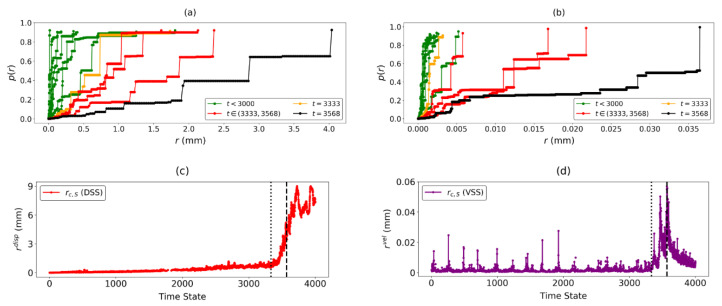
(Color online) Evolution of p(r) vs. *r* in (**a**) DSS and (**b**) VSS across different regimes: 1≤t≤3333 (pre-failure), $t^{*}=3333$ (regime change), 3333≤t≤3568 (landslide imminent), and $t_{F}=3568$ (landslide). The evolution with time of $r_{c,S}$ in both (**c**) DSS and (**d**) VSS. Dotted and dashed vertical lines refer to time states $t^{*}=3333$ and $t_{F}=3568$, respectively.

The separation of the predicted failure location $C^{*}$ from the rest of the rock slope is summarised by the p(r) vs. *r* profiles and the evolution of $r_{c,S}$ over time in both DSS and VSS. In both p(r) vs. *r* graphs (Figure 12a,b), the value of *r* preceding the formation of a system-spanning component increases in the lead up to failure, with a longer plateau in p(r), similar to our observations in B1 and B2. Compared to the evolution of p(r) with *r* in DSS, the rightward shift in the graphs in VSS is more distinct. This is consistent with our earlier result in Figure 10, where the predicted failure location $C^{*}$ from VSS provided a better signal of imminent failure. A sharp increase in the values of the two $r_{c,S}$ values is also observed at t=3333 (Figure 12c,d). This time state coincides with the increase in geographical similarity in the predicted failure location using $J_{t}^{\left(1\right)}$ and $J_{t}^{\left(2\right)}$, and reaffirms that $t^{*}=3333$ is the regime change point of the Mine. The increase in $r_{c,S}$ at $t^{*}$ can be interpreted in a similar fashion to B1 and B2, where the rigid-body-like motion between the failure and stable regions increases along the arcuate boundary of the landslide, the border between the stable zone and the slip region that collapses.

Altogether, our results suggest that the time when failure becomes imminent is $t^{*}=3333$. This corresponds to a date–time of 14 June, 13:16 p.m., approximately 24 h before the actual time of failure of 15 June, 13:10 p.m.

## 5. Discussion

We uncover a novel relationship between the evolution of the order of a percolation transition and the emergence of precursory signatures of localized failure in granular systems. This relationship proves robust for both laboratory and field scale conditions. We started with kinematic data across many times states from the stable regime through to failure: individual grain motion from simulations of laboratory tests on the one hand, and ground motion from real landslide monitoring on the other. At each time state, each data set forms a point pattern in the displacement state space. In the precursory failure regime, time-evolving clusters of densely packed points in displacement state space emerge, separated by low-density regions where points are relatively far apart from each other. This clustering pattern reflects the development of system partitions in physical state space, in which constituent grains move in unison, or, in the case of the rock slope, similar surface motions manifest at distinct pixel locations. We characterize the development of this collective motion in the corresponding network state space through the lens of explosive percolation. In particular, we tracked the time evolution of the network components as they approach the critical transition to explosive percolation and measured their corresponding critical radius $r_{c,S}$, the maximum separation among all the components in the network. In physical state space, these components give an early prediction of the ultimate pattern of failure for these systems, as previously studied using other techniques [1,2,9,28,31,32]. However, a time is eventually reached in the later stages of the precursory failure regime when the shared boundaries of these components persist in the same location across time. In systems with multiple shared boundaries (due to more than two components), different boundaries may manifest at different times but their respective locations do not change. During this period, a regime change point $t^{*}$ is reached when $r_{c,S}$, which quantifies the extent of relative sliding, undergoes a sudden and sharp increase to a peak. Thus, $t^{*}$ corresponds to when the partitions become incised in the physical state space, thereby precipitating the onset of slip between components along their shared boundaries. Moreover, the order parameter profile p(r) versus *r* at $t^{*}$ distinguishes itself from previous time states by a prolonged delay in percolation transition, as evident in a relatively long plateau preceding the jump.

With respect to findings from prior studies of the fully confined laboratory samples, $t^{*}$ coincides with the onset of collective buckling of columnar force chains, which rapidly culminate in the full development of the shear band at $t_{F}$ [26,29]. During $t^{*}\leq t\leq t_{F}$, the “broken pieces” of the granular body undergo relative sliding along their shared boundaries—the shear band (see also [3]). This explains the burst to a peak in the kinematic separation $r_{c,S}$. As shown in past studies of these systems, this time interval coincides with similarly sharp peaks in various measures of energy dissipation including: fluctuating kinetic energy, dissipation rate, average grain rotations, average local nonaffine deformation, and numbers of collapsing load-bearing structures (force chains, 3-cycles), and attendant vortices [26,28,29,30,32]. To test the robustness of the patterns, we quantified the spatiotemporal persistence of the shared boundary between the predicted components at consecutive time states in the lead up to the time of failure $t_{F}$.

In the field scale data on cumulative displacement for the rock slope, essentially the same trends were observed when compared to those from laboratory simulations. The interaction of the two unstable regions, the west wall and the southeast region, is reflected in the spatial variability of the predicted components for much of the precursory pre-failure regime. The instability along the west wall laterally shifts back and forth, but is mostly pulled towards the middle of the slope when the competing slide to the southeast is active. The complex dynamics of this interaction was recently characterized in [31]. To confirm this and the patterns uncovered, we carried out additional tests by repeating our analysis on the velocity data set, which is the information used to forecast the time of failure in landslide monitoring [15]. At the time of imminent failure $t^{*}$: the critical components of both state spaces converge to, and remain in, the same location in the physical state space, while their corresponding critical radii $r_{c,S}$ simultaneously undergo an abrupt increase to a peak. Moreover, the time of imminent failure is consistent with that recently uncovered in [31] using a combined statistical and machine learning approach. This highlights the need for future research into the optimal combination of kinematic properties for input data in forecasting the location and time of failure.

## 6. Conclusions

The concept of explosive percolation is used to develop a data-driven approach for characterization and prediction of granular failure from kinematic data. We test our approach using two data sets: one comprises individual grain motions from simulations of classical laboratory tests in which the granular material is driven to failure, the other from ground motions from radar monitoring of a developing landslide. Results demonstrate that our approach can provide an early prediction of the location of failure and help identify the time of imminent failure for both laboratory and field conditions. Accordingly, this work bridges a gap between fundamental knowledge gained from micromechanical studies of granular failure and the application of this knowledge in forecasting impending landslides. In the case of micromechanics, this study has also opened a path for future research into the influence of key material properties (such as grain shape and size distribution) on the overall strain localization pattern that demarcates the near-rigid body moving parts of the granular mass at failure. In the case of landslide forecasting, work is underway to explore the optimal combination of kinematic properties to analyze for a range of different landslides and to extend this approach to a probabilistic framework, which includes information on known triggers of landslides such as rainfall and seismic events.

Finally, our approach is general and can be employed to summarize the salient elements of a spatiotemporal dynamics of a system that develops a modular network structure or clustering pattern in a relevant feature state space. At variance with standard clustering algorithms (e.g., k-means used in [31]), the method proposed here does not need a pre-defined number of clusters for each point pattern. Also, the analysis can be extended to characterize the spatiotemporal dynamics in high dimensional feature state space and/or the spatial convergence of clustering structures derived from multiple lower dimensional feature state spaces.

## Figures and Tables

**Figure A1:**
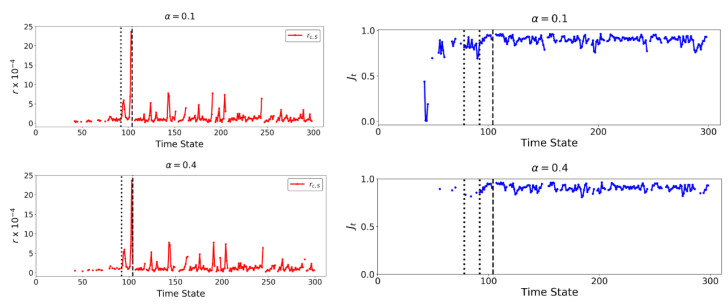
Evolution of $r_{c,S}$ and $J_{t}$ with time in the B1 sample for α=0.1 and α=0.4.

**Figure A2:**
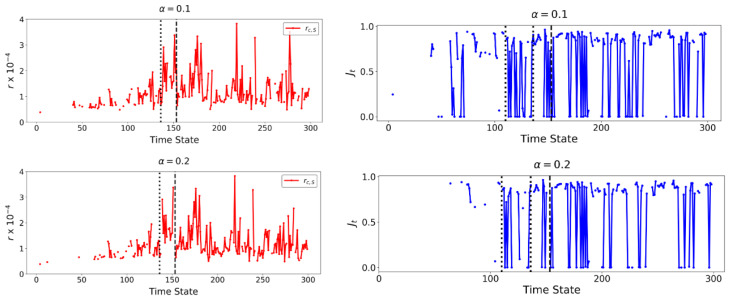
Evolution of $r_{c,S}$ and $J_{t}$ with time in the B2 sample for α=0.1 and α=0.2.

**Figure A3:**
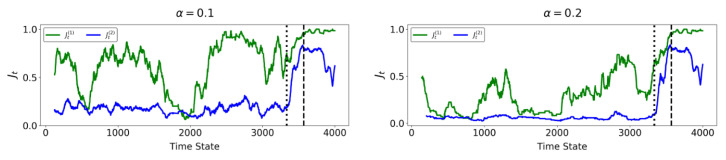
Evolution of $J_{t}^{\left(1\right)}$ and $J_{t}^{\left(2\right)}$ with time in the Mine for α=0.1 and 0.2.

## Appendix A. Glossary of Terms

**Table A1:** Glossary of terms.

Term	Definition
Physical state space (PSS)	The space defined by the geographical coordinates (x,y) of the pixels (grains).
Network state space (NSS)	The space in which the network is constructed.
Largest component	The component in the network that consists of the most number of nodes.
System-spanning component	A component that spans the network (greater than 80% of *N*).
Feature state space	The space defined by some feature of the pixels/grains in the system. It is *n*-dimensional depending on the feature chosen.
Displacement state space (DSS)	The space defined by the displacement of pixels (grains) in the system.
Velocity state space (VSS)	The space defined by the velocity of pixels (grains) in the system.
$d(C_{j},C_{k})$	The minimum distance between a node in one component, $C_{j}$ and a node in another component, $C_{k}$, in a feature state space.
$d(C_{k})$	The minimum inter-component distance of the component, $C_{k}$, which measures the separation of the component in the feature state space.
$r_{c,S}$	The critical radius or maximum separation across all components in the network.
$C^{*}$	The maximally separated component, i.e., $d\left(C^{*}\right)=r_{c,S}$.
$t^{*}$	Regime change point or time of imminent failure.
$t_{F}$	Time of failure or onset of failure regime.
$J_{t}$ or $J_{t}^{\left(1\right)}$	Similarity of failure pattern across consecutive time states using the same feature state space. The term $J_{t}^{\left(1\right)}$ is used if a secondary similarity measure ($J_{t}^{\left(2\right)}$) is considered.
$J_{t}^{\left(2\right)}$	Similarity of failure pattern across two feature state spaces, e.g., DSS and VSS, at the same time state.
Shared boundary	A collection of pairs of points, one from each of two distinct groups, that are in contact with each other in the physical state space.
α	A tuning parameter used to determine the minimum size for a component to be considered.
Discontinuous jump	A jump in the order parameter, characterised by the merger of a component to the current largest component in the network. We limit our attention to components that are of size greater than α=10% of *N*.

## Appendix B. Component Sizes

In this paper, we only consider components that are greater than α=0.1 of *N*. In B1, as two components form, we could retrospectively use a much higher value of α=0.4 to capture the major components in the sample. On the other hand for B2, where three components are present, an α value of 0.2 can be considered. In the following sets of figures, we show that the hyper-parameter α can act as a tuning parameter: at a higher value of α, only the larger blocks in the sample are identified, leading to more precise insights of a regime change point as long as the components that manifest near failure, including $C^{*}$, are not smaller than αN in size. Consequently, our graphs for $J_{t}$ are affected as smaller inconsequential components which form in the early precursory failure regime and are identified as $C^{*}$ but do not persist in the lead up to failure are no longer considered.

Figure A1 and Figure A2 below compare the different α profiles in B1–B2 respectively. In B1, the $J_{t}$ values at α=0.4 only appear frequently after $t^{*}=92$. As noted in Figure 6, in the time period t=78 to t=91, one of the two components that emerge is smaller than 0.4N in size. Only at $t^{*}=92$, does the shear band rotate marginally clockwise, whereby both components are greater than 0.4N in size and remain unchanged through to failure. In B2, the $J_{t}$ values for t<110 mostly disappear when α=0.2 and appear less frequently in the time interval t=110 to t=135. Essentially, the $J_{t}$ values fluctuate between 0 and 1 in the time states t=110 and t=120 and from t=136 onward. For both B1 and B2, $J_{t}$ is still defined for time states between $t^{*}$ and $t_{F}$ even at a larger value of α. This suggests that the evolution of the $r_{c,S}$ values in this time period remain unaffected too. Therefore, the regime change point $t^{*}$ does not change when a higher value of α is chosen. Instead, α could act as a tuning parameter that assists in the estimation of $t^{*}$.

We extend the analysis for the Mine in Figure A3 below by considering the impact of a higher α value on the similarity indices: $J_{t}^{\left(1\right)}$ and $J_{t}^{\left(2\right)}$. In the Mine, no more than three components emerge in DSS or VSS, so a conservative value of α=0.2 is considered. When α=0.2, the evolution of $J_{t}^{\left(1\right)}$ and $J_{t}^{\left(2\right)}$ is similar, with $J_{t}^{\left(2\right)}$ clearly increasing at $t^{*}=3333$. Meanwhile, $J_{t}^{\left(1\right)}$ fluctuates less and increases sharply towards 1 at $t^{*}=3333$. This supports our observation that both $J_{t}^{\left(1\right)}$ and $J_{t}^{\left(2\right)}$ approaches 1 at $t^{*}$, even at a higher α value.

## References

[1] Walker D., Tordesillas A., Pucilowski S., Lin Q., Rechenmacher A., Abedi S. (2013). Analysis of grain-scale measurements of sand using kinematical complex networks. Int. J. Bifurc. Chaos.

[2] Tordesillas A., Walker D.M., Rechenmacher A.L., Abedi S. (2011). Discovering community structures and dynamical networks from grain-scale kinematics of shear bands in sand. Advances in Bifurcation and Degradation in Geomaterials.

[3] Ma G., Regueiro R.A., Zhou W., Liu J. (2019). Spatiotemporal analysis of strain localization in dense granular materials. Acta Geotech..

[4] Nitka M., Tejchman J., Kozicki J., Leśniewska D. (2015). DEM analysis of micro-structural events within granular shear zones under passive earth pressure conditions. Granul. Matter.

[5] Tordesillas A., Walker D.M., Andò E., Viggiani G. (2013). Revisiting localized deformation in sand with complex systems. Proc. R. Soc. A Math. Phys. Eng. Sci..

[6] Fan X., Xu Q., Scaringi G., Dai L., Li W., Dong X., Zhu X., Pei X., Dai K., Havenith H.B. (2017). Failure mechanism and kinematics of the deadly June 24th 2017 Xinmo landslide, Maoxian, Sichuan, China. Landslides.

[7] Delacourt C., Allemand P., Berthier E., Raucoules D., Casson B., Grandjean P., Pambrun C., Varel E. (2007). Remote-sensing techniques for analysing landslide kinematics: A review. Bulletin de la Société Géologique de France.

[8] Zhao J., Wu J., Ding X., Mingzhou W. (2017). Elevation extraction and deformation monitoring by multitemporal InSAR of Lupu Bridge in Shanghai. Remote Sens..

[9] Tordesillas A., Zhou Z., Batterham R. (2018). A data-driven complex systems approach to early prediction of landslides. Mech. Res. Commun..

[10] Mistretta F., Sanna G., Stochino F., Vacca G. (2019). Structure from motion point clouds for structural monitoring. Remote Sens..

[11] Hu W., Hicher P.Y., Scaringi G., Xu Q., Van Asch T.W.J., Wang G. (2018). Seismic precursor to instability induced by internal erosion in loose granular slopes. Géotechnique.

[12] Piciullo L., Calvello M., Cepeda J.M. (2018). Territorial early warning systems for rainfall-induced landslides. Earth-Sci. Rev..

[13] Dick G.J., Eberhardt E., Cabrejo-Liévano A.G., Stead D., Rose N.D. (2015). Development of an early-warning time-of-failure analysis methodology for open-pit mine slopes utilizing ground-based slope stability radar monitoring data. Can. Geotech. J..

[14] Harries N., Noon D., Rowley K. (2006). Case Studies of Slope Stability Radar Used in Open Cut Mines.

[15] Intrieri E., Carlà T., Gigli G. (2019). Forecasting the time of failure of landslides at slope-scale: A literature review. Earth Sci. Rev..

[16] Stähli M., Sättele M., Huggel C., McArdell B.W., Lehmann P., Van Herwijnen A., Berne A., Schleiss M., Ferrari A., Kos A. (2015). Monitoring and prediction in early warning systems for rapid mass movements. Nat. Hazards Earth Syst. Sci..

[17] Casagli N., Catani F., Del Ventisette C., Luzi G. (2010). Monitoring, prediction, and early warning using ground-based radar interferometry. Landslides.

[18] Intrieri E., Bardi F., Fanti R., Gigli G., Fidolini F., Casagli N., Costanzo S., Raffo A., Di Massa G., Capparelli G. (2017). Big data managing in a landslide early warning system: Experience from a ground-based interferometric radar application. Nat. Hazards Earth Syst. Sci..

[19] Zhou Z., Tordesillas A. (2017). Powder keg divisions in the critical state regime: Transition from continuous to explosive percolation. EPJ Web Conf..

[20] Achlioptas D., D’Souza R.M., Spencer J. (2009). Explosive percolation in random networks. Science.

[21] Erdős P., Rényi A. (1960). On the evolution of random graphs. Publ. Math. Inst. Hung. Acad. Sci..

[22] D’Souza R.M., Gómez-Gardeñes J., Nagler J., Arenas A. (2019). Explosive phenomena in complex networks. Adv. Phys..

[23] Pan R.K., Kivelä M., Saramäki J., Kaski K., Kertész J. (2011). Using explosive percolation in analysis of real-world networks. Phys. Rev. E.

[24] Rozenfeld H.D., Gallos L.K., Makse H.A. (2010). Explosive percolation in the human protein homology network. Eur. Phys. J. B.

[25] Friedman E.J., Landsberg A.S. (2009). Construction and analysis of random networks with explosive percolation. Phys. Rev. Lett..

[26] Tordesillas A. (2007). Force chain buckling, unjamming transitions and shear banding in dense granular assemblies. Philos. Mag..

[27] Tordesillas A., O’Sullivan P., Walker D.M., Paramitha (2010). Evolution of functional connectivity in contact and force chain networks: Feature vectors, k-cores and minimal cycles. Comptes Rendus Mécanique.

[28] Tordesillas A., Pucilowski S., Walker D.M., Peters J.F., Walizer L.E. (2014). Micromechanics of vortices in granular media: Connection to shear bands and implications for continuum modelling of failure in geomaterials. Int. J. Numer. Anal. Methods Geomech..

[29] Walker D.M., Tordesillas A., Froyland G. (2014). Mesoscale and macroscale kinetic energy fluxes from granular fabric evolution. Phys. Rev. E.

[30] Tordesillas A., Pucilowski S., Lin Q., Peters J.F., Behringer R.P. (2016). Granular vortices: Identification, characterization and conditions for the localization of deformation. J. Mech. Phys. Solids.

[31] Das S., Tordesillas A. (2019). Near real-time characterization of spatio-temporal precursory evolution of a rockslide from radar data: Integrating statistical and machine learning with dynamics of granular failure. Remote Sens..

[32] Tordesillas A., Muthuswamy M., Walsh S.D. (2008). Mesoscale measures of nonaffine deformation in dense granular assemblies. J. Eng. Mech..

[33] D’Souza R.M., Nagler J. (2015). Anomalous critical and supercritical phenomena in explosive percolation. Nat. Phys..

[34] Bohman T., Frieze A., Wormald N.C. (2004). Avoidance of a giant component in half the edge set of a random graph. Random Struct. Algorithms.

[35] Chen W., Cheng X., Zheng Z., Chung N.N., D’Souza R.M., Nagler J. (2013). Unstable supercritical discontinuous percolation transitions. Phys. Rev. E.

[36] Ben-Hur A., Elisseeff A., Guyon I. (2002). A stability based method for discovering structure in clustered data. Pac. Symp. Biocomput..

